# Characterization of CRISPR Loci and Antimicrobial Resistance in Foodborne *Listeria monocytogenes* Isolates

**DOI:** 10.3390/microorganisms14071592

**Published:** 2026-07-21

**Authors:** Yufan Wu, Xiaoqiang Huang, Qiang Gu, Yun Li, Yuan Zhou, Lu Sun, Xiang Wang

**Affiliations:** 1 Centre of Analysis and Test, School of Chemistry & Molecular Engineering, East China University of Science and Technology, Shanghai 200237, China; wuyufan@ecust.edu.cn; 2 School of Health Science and Engineering, University of Shanghai for Science and Technology, Shanghai 200093, China; xl1509230513@outlook.com (X.H.); suvan7@163.com (Y.L.); zyuan0720@163.com (Y.Z.); sunlu_0707@163.com (L.S.); 3 Technology Center of Zhangjiagang Customs, Suzhou 215600, China; respro@163.com

**Keywords:** CRISPR arrays, antimicrobial resistance, *Listeria monocytogenes*, foodborne isolates

## Abstract

Clustered regularly interspaced short palindromic repeats (CRISPR) are widespread in bacterial and archaeal genomes as an adaptive immune system against invading mobile genetic elements. This study investigated the distribution of CRISPR loci and their potential association with antimicrobial resistance (AMR) in 40 foodborne *Listeria monocytogenes* isolates. CRISPR analysis showed that 18 isolates harbored CRISPR Locus 1, five carried Locus 2, and five possessed both loci. Antimicrobial susceptibility testing against seven antimicrobial agents indicated that most isolates were highly susceptible to the tested agents. Specifically, all isolates were susceptible to gentamicin, ampicillin, penicillin, and tetracycline, whereas resistance was observed in a small subset of isolates: four were resistant to chloramphenicol, five to levofloxacin, and four to ciprofloxacin. Statistical analysis showed no statistically significant association between the presence of CRISPR loci and antimicrobial susceptibility phenotypes. These findings provide baseline information on CRISPR locus distribution and antimicrobial susceptibility profiles in foodborne *L. monocytogenes* isolates. Further studies based on larger isolate collections, whole-genome sequencing, and characterization of associated *cas* genes are needed to clarify the potential role of CRISPR-Cas systems in AMR evolution in this species.

## 1. Introduction

CRISPR-Cas system represents an adaptive immune mechanism in prokaryotes, providing heritable protection against bacteriophages, plasmids, and other mobile genetic elements (MGEs) [1]. CRISPR locus was first observed in *Escherichia coli* as repeat sequences in 1987, and its formal designation as CRISPR was in 2002. The system has been identified in about 41% of bacterial genomes and 52% of archaeal genomes [2]. Structurally, a CRISPR-Cas locus typically comprises a CRISPR array containing conserved direct repeats separated by unique spacer sequences derived from past MGE encounters, as well as a set of *cas* genes encoding the protein machinery responsible for nucleic acid recognition and cleavage [3]. In addition to their primary role as adaptive immune systems that protect bacteria against invading foreign nucleic acids, CRISPR-Cas systems have increasingly been recognized for their broader functions in gene regulation, bacterial physiology and pathophysiology, virulence, and evolution [4].

*Listeria monocytogenes* is a major foodborne pathogen of global concern due to its ability to contaminate a wide range of ready-to-eat foods and persist in food processing environments [5]. Its exceptional tolerance to stressors such as refrigeration, high salt concentrations, and acidic pH enables survival during food storage and distribution [6]. Listeriosis, though relatively rare, poses a disproportionate threat to high-risk populations including the elderly, immunocompromised individuals, pregnant women, and newborns, and is associated with high hospitalization and fatality rates [7]. Despite this pathogenic potential, *L. monocytogenes* has historically shown susceptibility to a broad spectrum of antibiotics [6,8,9]. However, recent analyses have shown that the resistance rates of *L. monocytogenes* isolated from livestock and poultry meat in both China and the European Union have increased over the past two decades [10]. In addition, a recent analysis of national food surveillance data in China revealed that the detection rate of acquired multidrug resistance (MDR) in *L. monocytogenes* increased from 0.38% (11/2862) during 2012–2015 to 0.42% (23/5482) during 2016–2022 [11]. Although the acquired MDR rates remain low, the slight increase observed highlights the need for continuous surveillance to detect emerging resistance and prevent potential public health threats from new resistant clones [12].

Increasing reports of antibiotic-resistant *L. monocytogenes* isolates from food, animals, and clinical settings highlight the continued risk posed by horizontal gene transfer (HGT) [12]. HGT is a driving force in bacterial evolution, enabling the rapid dissemination of virulence factors, metabolic traits, and antimicrobial resistance determinants across species boundaries. CRISPR-Cas systems, which specifically target and degrade foreign nucleic acids, were originally proposed to restrict HGT and thereby limit the acquisition of antimicrobial resistance genes (ARGs) [1,13]. Early studies supported this view across several species: strains of *Streptococcus pneumoniae* lacking CRISPR loci acquired resistance more readily under selective pressure, loss of CRISPR elements in *Enterococcus faecalis* facilitated uptake of plasmid-mediated ampicillin resistance, the type I CRISPR system in *E. coli* interfered with the acquisition of drug-resistant plasmids (thereby preserving strain sensitivity), and a negative correlation between CRISPR-Cas presence and ARG acquisition was documented in *Klebsiella pneumoniae* [14,15,16]. However, more recent studies suggest that the relationship between CRISPR-Cas systems and antimicrobial resistance dissemination is far more complex than initially anticipated. In some species, such as *Staphylococcus aureus* [17,18], CRISPR-Cas systems are associated with, rather than opposed to, the spread of resistance genes, while studies in *E. coli* have shown weak or no consistent links between CRISPR loci and resistance phenotypes [19]. In addition, growing evidence indicates that CRISPR-Cas may, under certain conditions, facilitate horizontal gene transfer, particularly via phage-mediated transduction. For example, in *Pectobacterium atrosepticum*, CRISPR-Cas-mediated phage resistance increases transductant survival and thereby promotes the acquisition of non-phage genetic material [20]. These observations underscore the highly species-specific and context-dependent nature of the relationship among CRISPR-Cas systems, horizontal gene transfer, and antimicrobial resistance [16,21].

Despite the growing recognition of the ecological and evolutionary importance of CRISPR-Cas systems in bacteria, their relationship with antimicrobial resistance in *L. monocytogenes* remains largely unexplored. Given the significance of *L. monocytogenes* as a major foodborne pathogen and the increasing public health concern over emerging antimicrobial resistance, elucidating this relationship is of considerable importance. In this study, we examined the distribution of CRISPR loci and spacer diversity in a collection of foodborne *L. monocytogenes* isolates and explored their potential association with phenotypic antimicrobial susceptibility profiles. By combining CRISPR array identification, spacer analysis, and antimicrobial susceptibility testing, this work provides descriptive data to support further investigation of the possible role of CRISPR loci in antimicrobial variation in antimicrobial susceptibility in *L. monocytogenes*.

## 2. Materials and Methods

### 2.1. Bacterial Strains and Genomic DNA Extraction

A total of 41 *Listeria monocytogenes* strains were included in this study, comprising one reference strain (ATCC 19112) and 40 food-derived isolates. The 40 isolates were recovered from ready-to-eat foods collected in Shanghai between 2021 and 2023, including 38 from ready-to-eat meat products and 2 from ready-to-eat salads (Table S1). The detection of *L. monocytogenes* was performed according to GB 4789.30-2016 [22]. The food samples were homogenized in 225 mL of Listeria Enrichment Broth I (LB1, Guangdong Huankai Co., Ltd., Guangzhou, China) and enriched at 30 °C for 24 h. Then, 0.1 mL of the primary enrichment culture was transferred into 10 mL of Listeria Enrichment Broth II (LB2, Guangdong Huankai Co., Ltd., Guangzhou, China) for secondary enrichment under the same conditions. The enriched cultures were streaked onto Listeria Chromogenic Agar and PALCAM agar (Hopebiol Co., Ltd., Qingdao, China), followed by incubation at 36 °C for 48 h. Suspected *L. monocytogenes* colonies were further confirmed by biochemical tests [5]. *Staphylococcus aureus* ATCC 29213 was used as the quality control strain for antimicrobial susceptibility testing. All strains were preserved at −80 °C in 50% (*v*/*v*) glycerol. Prior to experimentation, frozen stocks were revived on tryptic soy agar supplemented with yeast extract (TSA-YE; Hopebio Co., Ltd., Qingdao, China) and incubated at 37 °C for 24 h, after which a single colony was inoculated into TSB-YE and cultured at 37 °C to obtain actively growing bacterial cultures. Genomic DNA was extracted using a DNeasy kit (Generay, Shanghai, China) according to the manufacturer’s protocol, and DNA concentration and purity were assessed using a NanoDrop™ 2000 spectrophotometer (Thermo Scientific, Waltham, MA, USA).

### 2.2. Primers and PCR Amplification

Three CRISPR loci in *L. monocytogenes* (Locus 1, Locus 2, and Locus 3) were amplified by PCR using previously described locus-specific primers (Table 1) [23]. PCR was carried out in a 25 μL reaction mixture containing 2 μL of template DNA, 12.5 μL of 2× PCR Master Mix (Generay, Shanghai, China), 1 μL of each primer, and 8.5 μL of molecular-grade water. The amplification conditions consisted of an initial denaturation step, followed by 35 cycles of denaturation at 95 °C for 30 s, annealing at 60 °C for 30 s, and extension at 72 °C for 1, 8, and 6 min for Locus 1, Locus 2, and Locus 3, respectively, with a final extension at 72 °C for 7 min. PCR products were resolved on 1.5% agarose gels, stained with GRgreen II (Generay Co., Ltd., Shanghai, China), and visualized using a gel documentation system (Biorad, Hercules, CA, USA). Positive amplicons were subjected to bidirectional Sanger sequencing by Sangon Biotech (Shanghai, China) using the same primers as those used for PCR amplification, to confirm the presence of CRISPR loci and determine the corresponding repeat and spacer sequences. Sanger sequencing was performed using single-primer linear amplification with fluorescently labeled dideoxynucleotides, followed by capillary electrophoresis and laser-based fluorescence detection. For bidirectional sequencing, forward and reverse sequencing reactions were conducted separately, the resulting sequences were assembled and editing was performed with the SeqMan module of the DNASTAR package (DNASTAR Inc., Version 7.1.0, Madison, WI, USA).

### 2.3. Identification of CRISPR Loci

CRISPR arrays were identified by the CRISPRs Finder web tool (https://crisprcas.i2bc.paris-saclay.fr/, accessed on 23 January 2026) [24]. Based on the multiple sequence alignment of repeat and spacer sequences, spacers with identical sequences or with less than 10% sequence divergence were classified as the same spacer type. Homology searches for each spacer were performed using CRISPRCasDb-Blast (https://crisprcas.i2bc.paris-saclay.fr/, accessed on 11 February 2026) [24]. Conservation analysis of the repeat sequences was conducted using WebLogo (https://weblogo.threeplusone.com/, accessed on 3 March 2026) [25].

### 2.4. Antibiotics Susceptibility Testing

Antimicrobial susceptibility testing was performed using the broth microdilution method according to the guidelines of the Clinical and Laboratory Standards Institute (CLSI) [26]. Minimum inhibitory concentrations (MICs) were determined for seven antimicrobial agents, including ampicillin (AMP), penicillin (PEN), gentamicin (GEN), tetracycline (TET), chloramphenicol (CHL), ciprofloxacin (CIP), and levofloxacin (LEVO) (Aladdin, Shanghai, China). Due to the limited availability of official CLSI criteria for *L. monocytogenes*, a dual-framework interpretation approach was adopted. Susceptibility profiles for AMP and PEN were interpreted based on the specific breakpoints provided in the CLSI M45 guideline. For the remaining five antibiotics (GEN, TET, CHL, CIP, and LEVO), which lack official *L. monocytogenes*-specific criteria in CLSI, the interpretive breakpoints established for *Staphylococcus* spp. were utilized as surrogate criteria (Table S2). Cross-resistance was defined as resistance to multiple antimicrobial agents within the same antibiotic class resulting from a shared resistance mechanism, while multidrug resistance (MDR) was defined as resistance to at least one agent in three or more antimicrobial classes. Briefly, single colonies of cultivated bacteria were suspended in sterile normal saline and adjusted to a 0.5 McFarland standard (equivalent to approximately 1.5 × 10^8^ CFU/mL). The bacterial suspension was then inoculated into Mueller-Hinton Broth (Haibo, Qingdao, China) to achieve a final structural concentration [27]. The prepared broths were subsequently dispensed into 96-well microtiter plates. After inoculation, the plates were incubated at 35 °C for 20–24 h before visual screening. *S. aureus* ATCC 29213 was utilized as the Gram-positive quality control strain to validate the accuracy of the MIC panels.

## 3. Results

### 3.1. Analysis of CRISPR Arrays

PCR amplification followed by agarose gel electrophoresis of 40 *L. monocytogenes* isolates revealed distinct bands corresponding to Locus 1 in 38 strains, whereas only two isolates showed no amplification. In contrast, distinct bands for Locus 2 were observed in six isolates, and no amplification products were detected for Locus 3 (Table S1). All successfully amplified PCR products were subjected to sequencing, and the resulting sequences were analyzed to confirm the presence and integrity of CRISPR arrays. Among the 40 *L. monocytogenes* isolates examined, CRISPR arrays were confirmed in 19 isolates (47.5%). Specifically, 18 isolates (45.0%) harbored Locus 1 and five strains (12.5%) harbored Locus 2, with four isolates carrying both loci. The lengths of the confirmed CRISPR arrays ranged from 223 to 613 bp for Locus 1 and from 609 to 684 bp for Locus 2.

### 3.2. CRISPR Repeat Sequence Analysis

**Figure 1:**
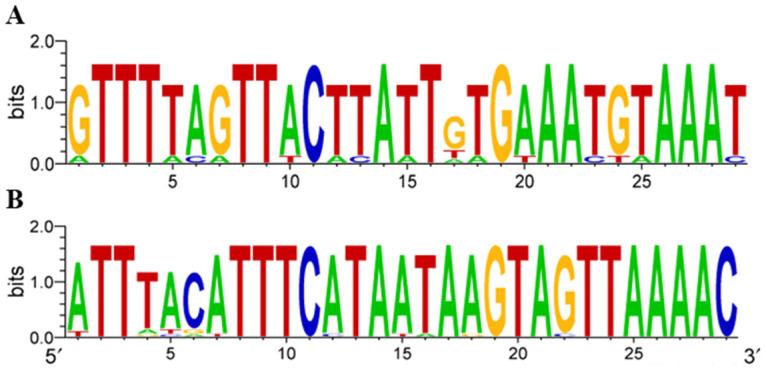
Sequence conservation of CRISPR repeat sequences in Locus 1 (**A**) and Locus 2 (**B**) of *L. monocytogenes*. Letter height reflects nucleotide conservation at each position.

Two repeat variants were identified in Locus 1, whereas a single repeat sequence was detected in Locus 2. All identified repeat sequences have been previously reported in the CRISPR database. WebLogo analysis of repeat sequence conservation showed that each repeat unit is 29 bp in length and is predominantly composed of A and T bases (Figure 1). Although repeat sequences within a CRISPR locus are typically highly conserved or even identical, repeats in Locus 1 showed lower conservation than those in Locus 2.

### 3.3. CRISPR Spacer Distribution Patterns

**Figure 2:**
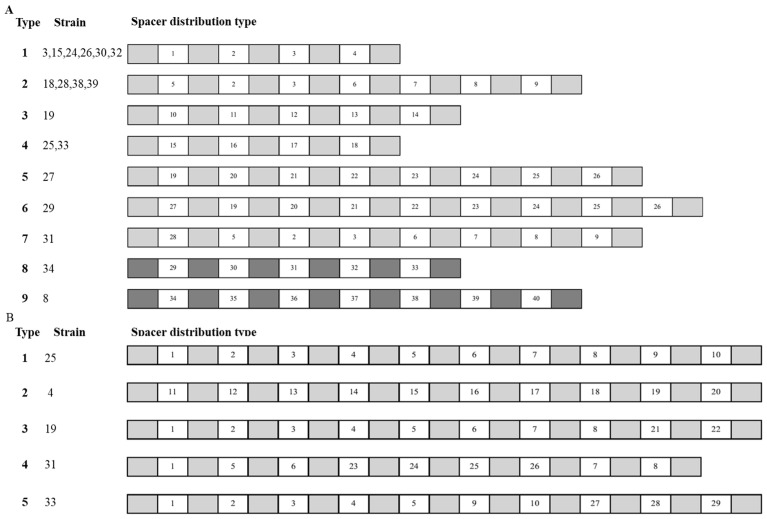
Spacer distribution types in Locus 1 (**A**) and Locus 2 (**B**) of *L. monocytogenes*. Grey and black blocks represent different repeat variants, and white blocks represent spacers. Numbers indicate spacer types within each panel; the same number in panels A and B does not indicate the same spacer sequence.

CRISPR arrays identified by CRISPRs Finder contained a total of 102 spacer sequences in Locus 1 and 49 spacer sequences in Locus 2. Multiple sequence alignment was performed for all spacer sequences, and spacers showing less than 10% sequence divergence were assigned to the same spacer type. Based on this criterion, 40 and 29 distinct spacer types were identified in Locus 1 and Locus 2, respectively (Figure 2). Spacer lengths ranged from 34 to 39 bp. Each CRISPR array exhibited a distinct spacer organization pattern. Among the 18 isolates harboring Locus 1, nine repeat-spacer distribution types were identified (Figure 2A), with types 1 and 2 being the most prevalent. In contrast, the five isolates containing Locus 2 were classified into five distinct types (Figure 2B). In Locus 1, the CRISPR arrays of types 2 and 7 were nearly identical, except that type 7 contained one additional repeat-spacer unit. In Locus 2, the CRISPR arrays of strains LM19 and LM25 were highly similar, differing only in the last two repeat-spacer units.

### 3.4. CRISPR Spacer Homology Analysis

To identify the putative origins of the spacers, BLAST searches were performed against multiple databases, including GenBank-Phage, RefSeq-Plasmid, GenBank-Viral, and IMG/VR v4. Of the 102 spacers identified in Locus 1, 79 (77.5%) showed significant matches to foreign nucleic acids. In Locus 2, 15 of 49 spacers (30.6%) had significant hits. The majority of matching spacers were homologous to phages and plasmids derived from *L. monocytogenes*. Representative results of the homology analysis are summarized in Table 2.

Some spacers from different isolates showed homology to the same plasmid, such as plasmid 5 of *L. monocytogenes* strain NCTC7974, suggesting that these isolates may have been exposed to related plasmid sequences. In addition, several spacers showed homology to multiple phages, including phages associated with *Listeria*, *Enterococcus*, *Streptococcus*, and *Bacillus* species. These findings indicate that the spacer content of *L. monocytogenes* reflects diverse historical exposures to mobile genetic elements. However, these homology results should be interpreted cautiously, as spacer matches alone cannot confirm direct horizontal gene transfer events.

### 3.5. Antimicrobial Susceptibility

Antibiotic susceptibility testing was performed on 40 *L. monocytogenes* isolates against seven antimicrobial agents (Table S1). All strains were fully susceptible to ampicillin (AMP), penicillin (PEN), gentamicin (GEN), and tetracycline (TET). Resistance to fluoroquinolones varied (Table 3). Only 4 strains (10.0%) were resistant to ciprofloxacin (CIP), while none showed intermediate resistance. In contrast, levofloxacin (LEVO) exhibited markedly lower efficacy: 29 strains (72.5%) were intermediately resistant, 5 strains (12.5%) were fully resistant, and only 6 strains (15.0%) remained susceptible. For chloramphenicol (CHL), 4 strains (10.0%) were resistant and 6 strains (15.0%) were intermediately resistant. Only two isolates showed resistance to two antimicrobial agents, both of which were resistant to LEVO and CIP. Overall, most *L. monocytogenes* isolates in this study remained susceptible to the tested antimicrobial agents, although a relatively high proportion of isolates showed reduced susceptibility to LEVO.

### 3.6. Correlation Between CRISPR Arrays and Antibiotics

The potential association between the presence of CRISPR loci (Locus 1 and Locus 2) and antimicrobial susceptibility phenotypes was investigated. Given the categorical nature of the variables and the limited number of resistant isolates, Fisher’s exact test was used for pairwise comparisons between the presence or absence of each CRISPR locus and susceptibility profiles to the seven tested antimicrobial agents. No statistically significant association was observed between CRISPR locus status and antimicrobial susceptibility (*p* > 0.05). Considering the limited number of resistant isolates and the uneven distribution of susceptibility categories, this association should be interpreted as preliminary.

## 4. Discussion

The CRISPR-Cas system functions as an adaptive immune mechanism that protects bacteria against invading phages and other mobile genetic elements. In addition to this canonical role, increasing evidence indicates that CRISPR-Cas systems also contribute to bacterial gene regulation, including the modulation of antimicrobial resistance, virulence, and biofilm formation [8,9]. CRISPR-Cas systems are classified into different types and subtypes mainly according to their associated *cas* genes, and type I and type II systems are the most frequently identified in *L. monocytogenes* [28]. Consistent with previous studies, Locus 2 identified in the present study belongs to subtype I-B, whereas the undetected Locus 3 corresponds to subtype II-A. Locus 1 represents a degenerate RliB locus lacking adjacent *cas* genes [29]. Sequence-based analysis confirmed the presence of Locus 1 in 17 isolates and Locus 2 in five isolates, whereas Locus 3 was not detected in any isolate.

The distribution and structural features of CRISPR arrays varied among the isolates. Although PCR amplification produced visible bands for Locus 1 in 38 of the 40 food-derived isolates and for Locus 2 in 6 strains, only 18 and 5 strains, respectively, were confirmed by sequence analysis to harbor complete CRISPR arrays. This discrepancy between PCR amplification and CRISPR array confirmation may be partly explained by the default criteria used in CRISPR Finder, which require at least three repeats with ≥80% sequence conservation for a confident CRISPR assignment [30]. Accordingly, some PCR products that generated visible bands were not classified as CRISPR arrays because they contained fewer than three repeats or exhibited insufficient repeat conservation. Consistent with this explanation, sequences derived from products without confirmed CRISPR arrays were generally shorter than those containing complete arrays. Consistent with this interpretation, Locus 1 showed lower repeat conservation and shorter array structures compared with Locus 2, supporting its degenerate nature. The number of spacers within each array also varied among strains, with smaller spacer numbers generally considered to reflect an earlier stage of CRISPR array evolution [31]. Notably, four strains (LM19, LM25, LM31, and LM33) carried both Locus 1 and Locus 2; no identical spacer sequences were observed between the two loci, indicating that these loci may record different histories of interaction with mobile genetic elements.

Antibiotic susceptibility testing of the 40 *L. monocytogenes* isolates against seven agents revealed uniformly high susceptibility to ampicillin (AMP), penicillin (PEN), gentamicin (GEN), and tetracycline (TET). Although tetracycline resistance has been reported previously in *L. monocytogenes*, the first resistant strain was reported in France in 1988 [32]. Studies have documented a low resistance level in TET [33,34], which is consistent with the findings of the present study. Ampicillin, administered alone or in combination with gentamicin, remains the first-line treatment for listeriosis [35], and all isolates in this study were fully susceptible to these key therapeutic agents. Resistance or reduced susceptibility was observed only in a small subset of isolates for chloramphenicol, levofloxacin, and ciprofloxacin. Overall, the low frequency of resistance among these foodborne isolates suggests that the tested population retained high susceptibility to several clinically relevant agents, although continued surveillance remains necessary. It is also worth noting that the interpretation of antimicrobial susceptibility in *L. monocytogenes* is constrained by the limited availability of species-specific breakpoints. Although CLSI and EUCAST provide guidance for many pathogen and antimicrobial combinations, interpretive criteria are not available for all foodborne pathogens or for all antimicrobial agents commonly tested in research. For *L. monocytogenes*, CLSI M45 provides criteria for only a limited number of clinically relevant agents, such as penicillin, ampicillin, trimethoprim-sulfamethoxazole, and meropenem. However, studies on foodborne *L. monocytogenes* often include broader antimicrobial panels. In the absence of *Listeria*-specific breakpoints, some studies have applied criteria established for related or clinically comparable organisms, such as *Staphylococcus* spp. [36] or *Bacillus* spp. [12]. In the present study, surrogate criteria for *Staphylococcus* spp. were applied where *L. monocytogenes*-specific criteria were unavailable. Although this approach provides a practical reference, it introduces uncertainty and may reduce comparability across studies. Differences between *L. monocytogenes* and *Staphylococcus* spp. in phylogenetic background, cell envelope properties, membrane permeability, and intrinsic resistance mechanisms may influence antimicrobial activity and MIC distributions.

Although the potential association between CRISPR-Cas systems and bacterial physiological traits, including antimicrobial resistance, has been widely proposed, relatively few studies have examined these relationships experimentally. In the present study, we investigated the association between CRISPR loci and antimicrobial susceptibility in *L. monocytogenes*. After reanalysis using Fisher’s exact test, no statistically significant association was observed between the presence of Locus 1 or Locus 2 and susceptibility to the tested antimicrobial agents. This result indicates that, within the present isolate collection, CRISPR locus status was not significantly associated with phenotypic antimicrobial resistance. The absence of significant associations should not be interpreted as definitive evidence that CRISPR-Cas systems have no relationship with antimicrobial resistance in *L. monocytogenes*. Rather, the limited sample size, the low prevalence of CRISPR loci, and the uneven distribution of resistance phenotypes reduced the statistical power to detect meaningful associations. In particular, most isolates were susceptible to the tested agents, and resistant isolates were observed only for a few antibiotics. Larger and more genetically diverse isolate collections will therefore be required to clarify whether CRISPR-Cas systems are associated with antimicrobial resistance patterns in this species.

In this study, CRISPR Locus 1 was identified in a subset of the *L. monocytogenes* isolates (17/40). Locus 1 is recognized as a degenerate RliB locus and lacks the associated *cas* operon required for canonical CRISPR-Cas immune activity [29]. Therefore, the presence of this locus should not be regarded as evidence of an active CRISPR-Cas system. This is consistent with the lack of significant association between Locus 1 and antimicrobial susceptibility profiles in the present study. More broadly, the functionality of a CRISPR-Cas system depends not only on the presence of CRISPR arrays but also on the integrity of the associated *cas* machinery, which is required for spacer acquisition, crRNA processing, and target interference. In this study, CRISPR characterization was based on targeted PCR amplification of CRISPR arrays, and the presence, integrity, and diversity of *cas* genes were not experimentally verified. Even when a CRISPR array is detected, mutations, frameshifts, or deletions in *cas* operons may impair system activity. Therefore, array detection alone provides only limited information on the functional capacity of CRISPR-Cas systems.

The relationship between CRISPR loci and antimicrobial resistance is complex and appears to be species- and context-dependent. CRISPR-Cas systems are generally considered barriers to horizontal gene transfer and may therefore limit the acquisition of plasmids or other mobile genetic elements carrying antimicrobial resistance genes [37,38]. However, previous studies have reported negative, weak, or even positive associations between CRISPR-Cas systems and antimicrobial resistance, depending on bacterial genetic background, CRISPR-Cas subtype, spacer-target matching, PAM recognition, and the functional integrity of associated *cas* genes [39,40,41]. In the present study, antimicrobial resistance was evaluated only at the phenotypic level, and the presence or absence of antimicrobial resistance genes was not further investigated. Although MIC determination provides important clinical and epidemiological information, it does not reveal the genetic determinants underlying resistance phenotypes. In the context of AMR, active CRISPR-Cas systems may influence resistance by restricting horizontal gene transfer. Therefore, correlating CRISPR arrays only with phenotypic susceptibility profiles provides an incomplete understanding of this relationship. The absence of whole-genome sequencing and comprehensive resistome analysis limited our ability to evaluate potential interactions between CRISPR loci, ARGs, and mobile genetic elements. Future studies should integrate phenotypic assays with WGS-based analyses, including ARG screening, plasmid profiling, and horizontal gene transfer assessment, to better clarify whether and how CRISPR-Cas systems shape the resistome of *L. monocytogenes*.

In summary, this exploratory study provides baseline information on the distribution of CRISPR arrays and phenotypic antimicrobial susceptibility profiles in foodborne *L. monocytogenes* isolates. Locus 1 and Locus 2 were detected in a subset of isolates, whereas Locus 3 was not observed. Although reduced susceptibility or resistance to several agents was detected in a small number of isolates, no statistically significant association was found between CRISPR locus status and antimicrobial susceptibility. These findings indicate that the relationship between CRISPR-Cas systems and antimicrobial resistance in *L. monocytogenes* cannot be resolved by phenotypic screening and CRISPR array detection alone. Larger studies integrating whole-genome sequencing, *cas* gene characterization, resistome analysis, and mobilome profiling are needed to clarify the potential role of CRISPR-Cas systems in the evolution of antimicrobial resistance in this pathogen.

## Figures and Tables

**Table 1:** Primers of CRISPR loci and conditions for PCR amplification.

CRISPR Loci	Sequence (5′–3′)	
Locus 1	F	TTGAGGTAAGATGGGAGTAAG
	R	ACAGATTGCTCGTTTGACTA
Locus 2	F	AAGGTGAAATAACTCCAGCA
	R	TTCGCCAATACCAAACTCG
Locus 3	F	GGGTCTATTTGGGCTGGTG
	R	GCGACGATTAGCGAGTTG

**Table 2:** Spacer sequence homology analysis of *L. monocytogenes*.

No. *	Spacer Sequences (5′–3′)	Homology Match
1	TGCTCTCTTTTGTGTAAAGCACATCAAGCATGTAGC	*L. monocytogenes* strain NCTC7974 plasmid 5
2	GCGATTTTTGTCAAAGGGACAGCGATGGGTTACAA	
3	TTACCACCAAAGTCCCTACACTCAATACCACCAAAGC	
4	AACAGAAATGAAAGATGGTGAAGAAGTCCTTAC	*Listeria* phage LP-013
5	AGTATTTACTTAGACAAACGCGCTCAAATTGAGCAT	*Enterococcus* phage phiFL4A
6	AAAATAGGAGGAAATAAATTATGACTATCAAATTAA	*Listeria* phage LP-101
		*Listeria* phage B025
7	TTTGTTGAATCAACGGATATAGATTTTACAATTTC	*Listeria* phage A006
		*L. monocytogenes* strain NCTC7974 plasmid 5
8	GAAATCGAGGTGGTTTGATGCCGATTAAAGTACGTGA	*Listeria* phage vB_LmoS_293
9	ATATTTGACCGTGCCCGGTAAAACTACCGCAAACGT	*Listeria* phage LP-030-2
10	TAAGATGTCCGATAATGAACACGCGTTCTCTGTTTTG	*Listeria* phage vB_LmoS_293
		*Streptococcus* phage P0094
		*Lactococcus* phage CHPC1175
		*Bacillus* phage vB_BhaS-171

* Numbers 1 to 7 are spacers of Locus 1; 8 to 10 are spacers of Locus 2.

**Table 3:** Antimicrobial resistance of *L. monocytogenes*.

Antibiotics	Antimicrobial Resistance		
	Sensitive ^1^	Intermediate ^1^	Resistant ^1^
Ciprofloxacin	90.0% (36)	0% (0)	10.0% (4)
Levofloxacin	15.0% (6)	72.5% (29)	12.5% (5)
Ampicillin	100% (40)	0% (0)	0%
Penicillin	100% (40)	0% (0)	0%
Gentamicin	100% (40)	0% (0)	0%
Chloramphenicol	75.0% (30)	15.0% (6)	10.0% (4)
Tetracycline	100% (40)	0% (0)	0%

^1^ Data are expressed as the number of strains and the corresponding percentages (%).

## Data Availability

The original contributions presented in this study are included in the article/Supplementary Materials. Further inquiries can be directed to the corresponding author.

## Supplementary Materials

The following supporting information can be downloaded at: https://www.mdpi.com/article/10.3390/microorganisms14071592/s1, Table S1. Source, CRISPR locus distribution, and antimicrobial susceptibility profiles of foodborne *L. monocytogenes* isolates; Table S2. Interpretive criteria for antimicrobial susceptibility testing in *L. monocytogenes*.
