# Supplementary material for: Characterization of CRISPR Loci and Antimicrobial Resistance in Foodborne Listeria monocytogenes Isolates
Source: Microorganisms. 2026 Jul 21;14(7):1592. doi: 10.3390/microorganisms14071592 (PMC13414327; doi:10.3390/microorganisms14071592)
Supplement: Supplementary file 1 [file microorganisms-14-01592-s001.zip › microorganisms-4311287-supplementary.pdf]

Table S1. Source, CRISPR locus distribution, and antimicrobial susceptibility profiles of foodborne *L. monocytogenes* isolates

Strain	Source	Locus 1	Locus 2	AMP	PEN	GEN	TET	CHL	CIP	LEVO
LM1	RET meat products	-	-	S	S	S	S	R	S	I
LM2	RET meat products	-	-	S	S	S	S	R	S	I
LM3	RET meat products	+	-	S	S	S	S	R	S	I
LM4	RET meat products	-	+	S	S	S	S	S	S	I
LM5	RTE salad	-	-	S	S	S	S	S	S	S
LM6	RTE salad	-	-	S	S	S	S	I	S	S
LM7	RET meat products	-	-	S	S	S	S	S	S	I
LM8	RET meat products	+	-	S	S	S	S	S	S	I
LM9	RET meat products	-	-	S	S	S	S	I	S	I
LM10	RET meat products	-	-	S	S	S	S	S	S	S
LM11	RET meat products	-	-	S	S	S	S	S	S	I
LM12	RET meat products	-	-	S	S	S	S	S	S	I
LM13	RET meat products	-	-	S	S	S	S	I	S	I
LM14	RET meat products	-	-	S	S	S	S	S	S	I
LM15	RET meat products	+	-	S	S	S	S	S	S	I
LM16	RET meat products	-	-	S	S	S	S	S	S	R
LM17	RET meat products	-	-	S	S	S	S	I	S	R
LM18	RET meat products	+	-	S	S	S	S	R	S	I
LM19	RET meat products	+	+	S	S	S	S	S	S	I
LM20	RET meat products	-	-	S	S	S	S	S	R	R
LM21	RET meat products	-	-	S	S	S	S	S	S	I
LM22	RET meat products	-	-	S	S	S	S	S	S	I
LM23	RET meat products	-	-	S	S	S	S	S	S	S

LM24	RET meat products	+	-	S	S	S	S	I	S	I
LM25	RET meat products	+	+	S	S	S	S	S	S	S
LM26	RET meat products	+	-	S	S	S	S	S	R	I
LM27	RET meat products	+	-	S	S	S	S	S	S	S
LM28	RET meat products		-	S	S	S	S	S	S	I
LM29	RET meat products	+	-	S	S	S	S	S	S	I
LM30	RET meat products	+	-	S	S	S	S	S	S	I
LM31	RET meat products	+	+	S	S	S	S	S	S	I
LM32	RET meat products	+	-	S	S	S	S	S	R	R
LM33	RET meat products	+	-	S	S	S	S	S	S	I
LM34	RET meat products	+	-	S	S	S	S	S	R	I
LM35	RET meat products	-	+	S	S	S	S	I	S	R
LM36	RET meat products	-	-	S	S	S	S	S	S	S
LM37	RET meat products	-	-	S	S	S	S	S	S	I
LM38	RET meat products	+	-	S	S	S	S	S	S	S
LM39	RET meat products	+	-	S	S	S	S	S	S	I
LM40	RET meat products	-	-	S	S	S	S	S	S	I

Note: RTE, ready-to-eat; “+” indicates the presence of the corresponding CRISPR locus, whereas “-” indicates its absence. AMP, ampicillin; PEN, penicillin; GEN, gentamicin; TET, tetracycline; CHL, chloramphenicol; CIP, ciprofloxacin; LEVO, levofloxacin. S, susceptible; I, intermediate; R, resistant.

Table S2. Interpretive criteria for antimicrobial susceptibility testing in *L. monocytogenes*

Antimicrobial Agent (μg/mL)	Susceptible (S)	Intermediate (I)	Resistant (R)
Ampicillin	≤ 2	-	-
Penicillin	≤ 2	-	-
Gentamicin	≤ 4	8	≥ 16
Tetracycline	≤ 4	8	≥ 16
Chloramphenicol	≤ 8	16	≥ 32
Ciprofloxacin	≤ 1	2	≥ 4
Levofloxacin	≤ 1	2	≥ 4

Note: According to the CLSI guidelines, breakpoints for ampicillin and penicillin were provided. For all other compounds, the reference breakpoints for *Staphylococcus* spp. were interpreted.
